# Serum levels of anti-Müllerian hormone influence pregnancy outcomes associated with gonadotropin-releasing hormone antagonist treatment: a retrospective cohort study

**DOI:** 10.1038/s41598-023-28724-8

**Published:** 2023-02-06

**Authors:** Yanru Hou, Lu Wang, Yian Li, Jiajia Ai, Li Tian

**Affiliations:** 1 Reproductive Medicine Center, Department of Obstetrics and Gynecology, Peking University People’s Hospital, Peking University Health Science Center, Beijing, China; 2 Gynaecology and Obstetrics, Beijing Jishuitan Hospital, Beijing, China

**Keywords:** Predictive markers, Endocrinology, Medical research

## Abstract

As a specific predictor of ovarian reserve, serum anti-Müllerian hormone (AMH) has become an area of intense research interest in the field of assisted reproductive technology. We assessed the relationship between AMH levels and pregnancy outcomes in Chinese patients and investigate the influencing factors of cumulative live birth in patients with high AMH levels. A total of 1379 patients starting their IVF/ICSI cycle were divided into normal (Group A, 1.1–4.0 ng/ml, n = 639) and high (Group B, > 4.0 ng/ml, n = 740) groups by serum AMH levels. Live birth rate (LBR), cumulative live birth rate (CLBR) and cumulative clinical pregnancy rate (CCPR) were also investigated. Compared with Group A, Group B had a significantly higher CLBR (65.80% vs. 43.95%) and CCPR (76.77% vs. 57.14%), respectively. Binomial logistic regression analysis showed that age over 40 years, LH/FSH > 2.5, total Gn dose and Gn duration, and greater than 4000 ng/ml serum E2 levels on HCG day were significantly associated with CLBR in Group B. The AUC value of CLBR averaged 0.664 (ranging from 0.621 to 0.706) (*p* < 0.001). The patients with high AMH levels had higher CPR, higher LBR, and lower MR with no statistically significant differences, although there were significant improvements in CLBR. Advanced age (> 40 years) still impacted CLBR, even in women with good ovarian reserves. Consequently, it is still recommended that patients over 40 years old with high AMH levels actively receive IVF treatment if they seek to become pregnant. PCOS diagnoses did not influence the CLBR. In summary, this study showed that serum AMH levels could positively predict patient ovarian responses and further affect pregnancy outcomes.

## Introduction

Ovarian reserves play an important role in predicting in vitro fertilization (IVF) /Intracytoplasmic sperm injection (ICSI) outcomes^1^. Previous studies have used the ovarian reserve markers antral follicle count (AFC), follicle stimulating hormone (FSH), and anti-Mullerian hormone (AMH)^1^ for this purpose. However, it remains unclear which is the most sensitive marker of ovarian reserve. Over time, as more relevant studies have been published, the classic joint markers of AFC and AMH have been replaced by AMH alone^1,2^. In recent years, AMH has become an area of intense research interest in the field of assisted reproductive technology (ART). AMH is a glycoprotein dimer that belongs to the transforming growth factor-β (TGF-β) superfamily; it is produced by granulosa cells (GCs) in the preantral, antral, and preovulatory follicles in the ovary^3,4^. AMH prevents follicular growth by inhibiting aromatase and downregulating luteinizing hormone receptor until these follicles are stimulated by other factors^5^. Compared to AFC and FSH, AMH is more reliable as an indicator of ovarian reserve because it shows minimal variation between menstrual cycle stages, menstrual cycles, and individuals^4,6–9^. Because of this consistency, AMH has become a widely-used tool for assessing ovarian reserves^6,10^. It has also been studied as a predictor of potential oocyte production before ovulation induced by ART. One study aiming to demonstrate a reliable correlation between AMH and oocyte production showed that patients with 11 or more oocytes in the ART cycle had 2.5-fold higher AMH levels than patients with six or fewer oocytes. This correlation between ART success and AMH extended to the live birth rate (LBR); a retrospective study observed the results of 1230 IVF-ICSI cycles and found that the probability of live birth in those with AMH ≥ 2.94 ng/ml increased in a logarithmic manner^11^. Many studies have confirmed the correlation between higher AMH levels and better ART outcomes^12–14^. However, other studies have shown that AMH levels > 3.5 ng/ml are not associated with significant increases in LBR^15–18^. The true relationship between AMH levels and LBR is thus controversial. With the widespread application of efficient embryo freezing technologies, the cumulative live birth rate (CLBR), including the LBR of fresh embryo transfer and subsequent frozen embryo thawing transfer (FET) after a single ovarian stimulation cycle, has gradually come to be considered an important indicator of ART success^19^. In practice, infertile couples may have to undergo several IVF/ICSI attempts to reach the desired outcome, and their expected prognosis is based on the chance of success within a number of years from the start of treatment^20^. However, to date, no study has comprehensively explored the factors that influence CLBR after IVF/ICSI in patients with high AMH levels. Therefore, this study was conducted to evaluate the relationship between serum AMH levels and pregnancy outcomes in Chinese patients receiving IVF/ICSI, and to investigate the factors influencing CLBR in patients with high AMH levels.

## Methods

### Participant selection

This was a retrospective, single-center cohort study. We collected clinical data for 1379 patients who received the gonadotropin-releasing hormone (GnRH) antagonist protocol at the Reproductive Medicine Center of Peking University People’s Hospital between January 2016 and December 2020. Exclusion criteria included: (1) serum AMH levels < 1.1 ng/ml; (2) a diagnosis of hypothalamic amenorrhea; (3) uterine disease, including uterus submucous myoma, uterine mediastinum, uterine adhesions, a thin endometrium, and immature uterus. Serum AMH concentrations were determined using a UniCel DxI 800 Access automatic chemiluminescence immunoassay instrument (Beckman Coulter, Brea, CA, USA) and an AMH microparticle enzymatic luminescence kit (Beckman Coulter). Patients were divided into two groups based on serum AMH levels: normal (Group A, 1.1–4.0 ng/ml; n = 639) and high (Group B, > 4.0 ng/ml; n = 740). Other patient data collected included age, body mass index (BMI), duration of infertility, polycystic ovary syndrome (PCOS) status, baseline follicle-stimulating hormone (bFSH) levels, baseline luteinizing hormone (bLH) levels, AFC levels, initial gonadotropins (Gn) dose, total Gn dose, duration of Gn treatment, serum concentrations of estradiol (E2), progesterone (P), and luteinizing hormone (LH) on human chorionic gonadotrophin (HCG) day, the number of retrieved oocytes, and the number of metaphase II (MII) oocytes. Endpoint measurements included the clinical pregnancy rate (CPR), early miscarriage rate (MR), LBR, cumulative clinical pregnancy rate (CCPR), and CLBR. PCOS was classified based on the presence of two of the three 2003 Rotterdam PCOS criteria: (1) oligo- or anovulation; (2) clinical and/or biochemical signs of hyperandrogenism; and (3) polycystic ovaries combined with the exclusion of other etiologies (e.g., congenital adrenal hyperplasia, androgen-secreting tumors, or Cushing’s syndrome)^21^. This study was approved by the Ethics Committee of Peking University People’s Hospital (reference number 2021PHB083-001) and performed in accordance with the Declaration of Helsinki. All participants provided informed consent.

### IVF/ICSI procedure

The baseline values of FSH, LH, E2, and P were measured. All patients received a vaginal ultrasound on the second day of menstruation, except those with bilateral ovarian cysts or pregnancy. The initial dose of r-FSH (Gonal-F; Merck Serono Biopharma) was determined based on maternal age, BMI, and levels of AFC, bFSH, and AMH. Beginning on the second day of the menstrual cycle, all patients received r-FSH at an initial dose of 150–300 IU for 4 or 5 d. The daily dose was adjusted based on the ovarian response. When the diameter of one follicle reached ≥ 13–14 mm, a GnRH antagonist (ganirelix [Organon] or cetrorelix acetate [Merck Serono Biopharma]) was administered. When at least two leading follicles reached 18 mm in diameter, 250 μg recombinant human chorionic gonadotropin (rhCG) (Ovidrel, Merck Serono Biopharma) was administered to induce oocyte maturation. Oocytes were retrieved 36 h later. Embryos were then classified according to morphological criteria. Day 3 embryos had at least six to 10 cells and a fragmentation rate < 20%. Blastocysts showed a distinct trophectoderm and inner cell mass on Day 5^22^. All embryos were transferred on the third or fifth day after egg retrieval. The number of embryos transferred (one or two) was determined based on maternal age and embryo quality. Patients received progesterone supplements for luteal phase support. Individuals with serum β-HCG levels > 5 IU/ml at 14 d after embryo transfer were considered pregnant. Clinical pregnancy was defined as having an intrauterine sac detected by ultrasonic examination at 4 weeks after embryo transfer. Early miscarriage was defined as a loss of clinical pregnancy before 12 weeks, a lack of fetal heart activity, or disappearance of fetal heart activity. LBR was defined as the delivery of one live baby after the 28th week of pregnancy^22^. For all cases of clinical pregnancy, luteal phase support continued until the 12th week of gestation. Patients who were not pregnant after a fresh embryo transfer could undergo frozen-thawed replacement cycles in either a natural or an artificial cycle.

### Outcome measurements

The main outcome measured was CLBR, which was defined as the number of deliveries with at least one live birth resulting from one initiated ART cycle. For each individual, this comprised a one-year treatment period including all cycles in which fresh and/or frozen embryos were transferred, until either one delivery with a live birth occurred or all embryos were used. CCPR was defined as the total number of clinical pregnancies arising from fresh or frozen embryo transfer following one initiated ART cycle per patient during the one-year treatment period^23–25^.

### Data analysis

The required sample size for statistical power at 99% with a two-sided alpha level of 0.05 and a dropout rate of 10% was calculated using PASS v15.0. The actual sample sizes (639 in group A and 740 in group B) achieved 99% power to detect differences between the groups at a proportion of − 0.2185. The proportion in group A was assumed to be 0.6580 under the null hypothesis and 0.4395 under the alternative hypothesis. The proportion in group B was 0.6580. The test statistic used was the two-sided Z-test with unpooled variance. The significance level of the test was 0.050.

Statistical Package for the Social Sciences (SPSS, v19.0) was used to perform statistical analyses. Continuous variables were analyzed by calculating the mean and standard deviation (SD). Normal and nonnormal distributions were analyzed using independent *t*-tests and Mann–Whitney *U* tests, respectively. In comparisons between two groups, significant differences were explored using the Bonferroni correction method to obtain an α value. Categorical variables were compared using a chi-square test or a Fisher’s exact test and presented as raw frequencies with corresponding percentages; a *p*-value of < 0.05 was considered statistically significant. The potential factors associated with pregnancy outcomes were analyzed with a binary logistic regression model. An odds ratio (OR) with 95% confidence intervals (CIs) indicated the strength of relevance. A receiver operating characteristic (ROC) curve analysis was performed to evaluate the predictive ability of AMH levels for CLBR and CCPR. The area under the ROC curve (AUC) was calculated to judge the predictive value of these influencing factors for CLBR and CCPR in patients with high AMH levels^26^.

### Ethics approval and consent to participate

This study was approved by the Ethics Committee of Peking University People's Hospital (reference number 2021PHB083-001) and performed in accordance with the Declaration of Helsinki. All participants provided informed consent.

## Results

Significant differences in age, duration of infertility, PCOS, bFSH, bLH, AFC, total Gn dose , Gn duration, serum E2, P, and LH levels on HCG day, the numbers of retrieved oocytes and MII oocytes were noted in Group B compared with Group A. Compared with Group A, Group B exhibited the following features: younger maternal age, shorter duration of infertility, more PCOS patients, lower bFSH, higher bLH, more AFC, longer Gn duration, less total Gn dose, higher E2 levels on HCG day, greater number of retrieved oocytes and MII oocytes (Tables 1 and 2).

**Table 1:** Baseline characteristics for the total group.

	Group A (AMH = 1.1–4.0 ng/ml)	Group B (AMH > 4.0 ng/ml)	*p* value
Number	639	740	
Age(years)	34.24 ± 4.21	31.67 ± 4.08	< 0.001
BMI(kg/m2)	22.83 ± 4.46	23.28 ± 7.86	0.205
Duration of infertility(years)	3.80 ± 3.02	3.40 ± 2.66	0.009
PCOS (%)	24(3.76%)	175(23.65%)	< 0.001
Basal FSH(IU/ml)	7.81 ± 2.45	6.70 ± 1.91	< 0.001
Basal LH(IU/ml)	4.02 ± 1.96	5.07 ± 2.87	< 0.001
AMH(ng/ml)	2.29 ± 0.80	7.10 ± 3.88	< 0.001
AFC(n)	9.23 ± 4.28	15.93 ± 6.83	< 0.001
Initial Gn dose(IU)	261.69 ± 79.62	187.92 ± 57.27	< 0.001
Gn Duration(-days)	9.28 ± 1.65	9.48 ± 1.68	0.029
Total Gn dose (IU)	2654.14 ± 908.48	1896.41 ± 689.52	< 0.001
Endometrial thickness(cm)	0.98 ± 0.26	0.98 ± 0.33	0.871
E2 on HCG day(ng/ml)	1981.56 ± 1289.64	2999.74 ± 1525.09	< 0.001
LH on HCG day(mIU/ml)	3.43 ± 3.67	3.12 ± 2.98	< 0.001
P on HCG day(ng/ml)	1.21 ± 1.30	1.40 ± 1.37	0.009

**Table 2:** Cycle characteristics and ART outcomes.

	Group A (AMH = 1.1–4.0 ng/ml)	Group B (AMH > 4.0 ng/ml)	*p* value
Retrieved oocytes(n)	9.83 ± 5.31	16.40 ± 8.12	< 0.001
MII oocytes(n)	8.48 ± 4.84	14.04 ± 7.44	< 0.001
Fresh embryo transfer(n)	259	160	
CPR	44.79%(116/259)	48.13%(77/160)	0.545
LBR	37.07%(96/259)	41.25%(66/160)	0.603
MR	7.34%(19/259)	6.25%(10/160)	0.695
CCPR	57.14%(232/406)	76.77%(476/620)	< 0.001
CLBR	43.95%(178/405)	65.80%(406/617)	< 0.001

**Figure 1:**
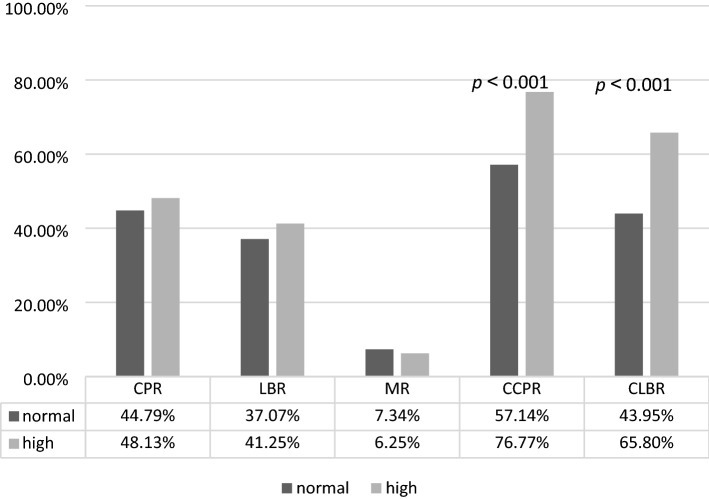
CPR, LBR and MR per embryo transfer as well as CCPR and CLBR in the serum AMH concentration groups on day 2. Except for MR, other parameters increased from normal to high in the AMH groups. Moreover, CCPR and CLBR had significant differences.

**Figure 2:**
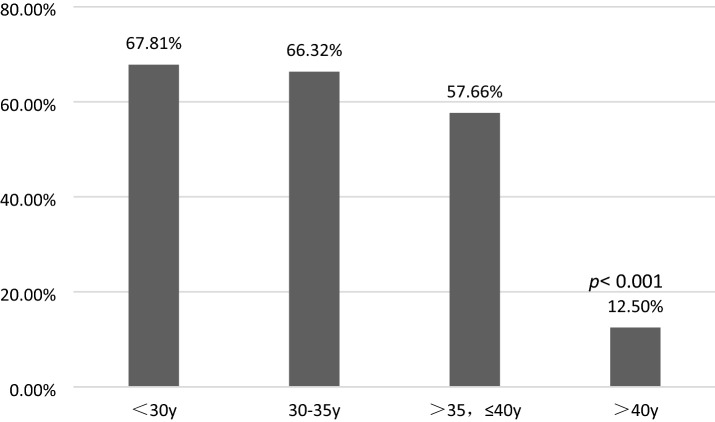
With maternal age increased, CLBR of high AMH levels patients decreased. Particularly, CLBR of over 40 years old patients dropt dramatically with significant difference than other subgroups.

**Figure 3:**
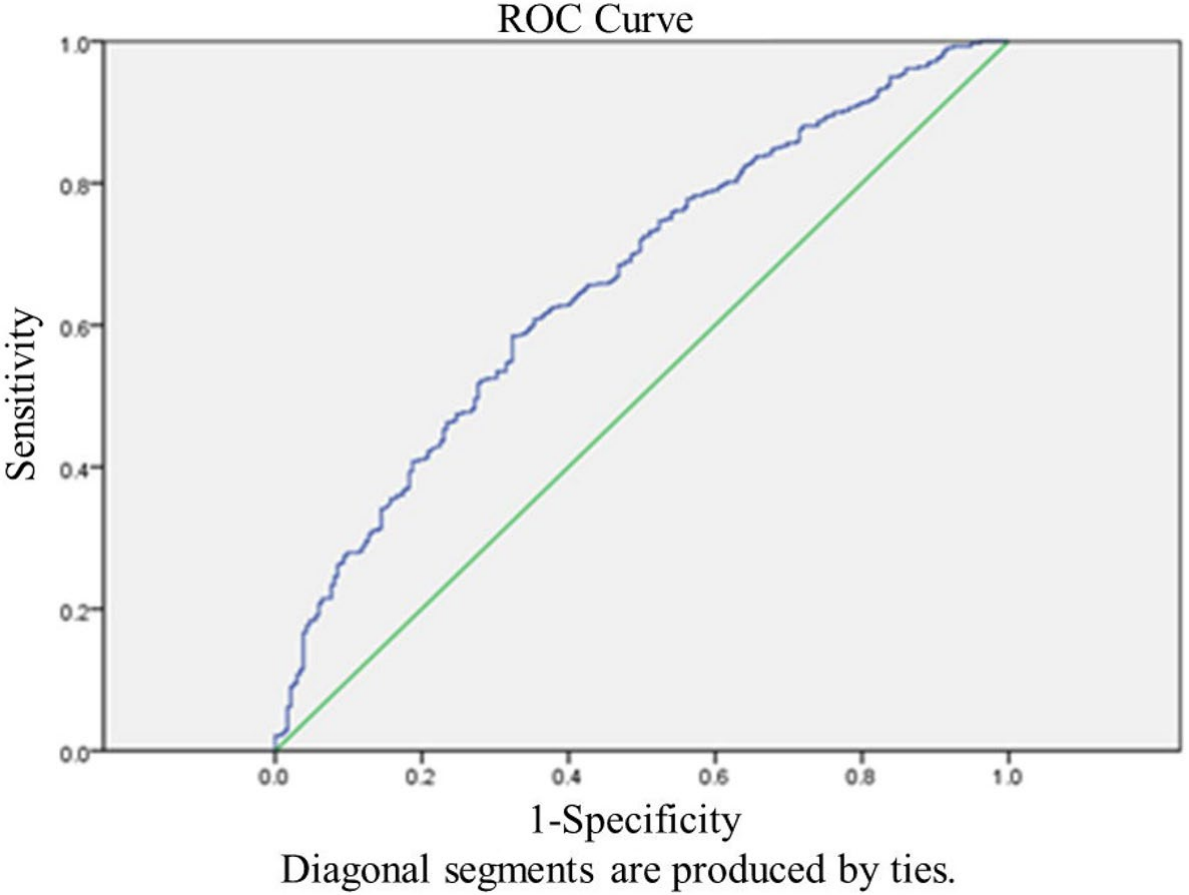
Receiver-operating characteristic (ROC) curve analysis for AMH as a predictor of CLBR. The AUC of CLBR averaged 0.664 (ranging from 0.621–0.706), *p* < 0.001.

**Figure 4:**
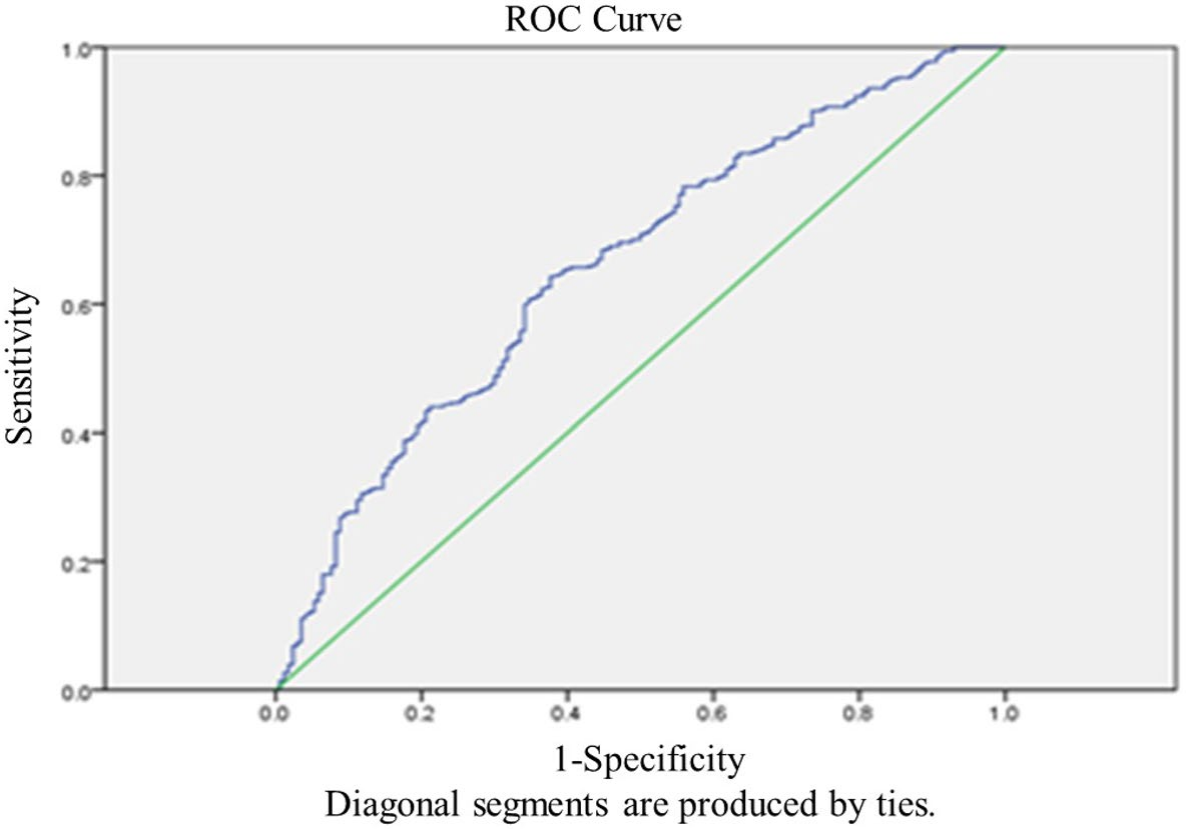
Receiver-operating characteristic (ROC) curve analysis for AMH as a predictor of CCPR. The AUC of CCPR averaged 0.661 (ranging from 0.613– 0.708), *p* < 0.001.

The age range of all the patients is 21–45 years. And the age range of normal AMH and high AMH patients are 21–45 years and 24–44 years. No significant differences were noted between Group A and Group B at LBR (37.07% vs. 41.25%, respectively) and CPR (44.79% vs. 48.13%, respectively). Compared with Group A, Group B had a significantly higher CLBR (65.80% vs. 43.95%, respectively) and CCPR (76.77% vs. 57.14%, respectively) (Table 2, Fig. 1). Binomial logistic regression analysis showed that age greater than 40 years, LH/FSH > 2.5, total Gn dose, Gn duration, and E2 levels greater than 4000 ng/ml on HCG day were significantly associated with CLBR in Group B (Table 3, Fig. 2). The AUC values of CLBR and CCPR averaged 0.664 (ranging from 0.621 and 0.706) and 0.661 (ranging from 0.613 and 0.708) (*p* < 0.001), respectively, demonstrating moderate predictive value of AMH levels for CLBR and CCPR. (Figs. 3 and 4).

**Table 3:** The multivariate logistic regression analysis of confounding factors for CLBR in Group B.

Factor	Odds ratio(95% CI)	*p* value
Age,y		
< 30		
30 ≤ age ≤ 35	0.963(0.655–1.415)	0.846
35 < age ≤ 40	0.633(0.375–1.066)	0.086
> 40	0.137(0.028–0.680)	0.015
BMI, kg/m^2^	0.971(0.862–1.094)	0.629
< 18		
18–24	0.691(0.299–1.597)	0.387
> 24, ≤ 28	0.561(0.230–1.367)	0.203
> 28	0.471(0.178–1.242)	0.128
Primary/Secondary infertility	1.080(0.764–1.527)	0.664
PCOS(n)	0.735(0.471–1.147)	0.175
bFSH(IU/ml)	1.066(0.469–2.421)	0.879
< 10		
≥ 10		
bLH/bFSH	0.092(0.010–0.881)	0.039
≤ 2.5		
> 2.5		
AFC(n)		
< 7		
7–20	1.589(0.768–3.286)	0.212
> 20	1.681(0.744–3.796)	0.212
Initial Gn dose(IU)		
< 150		
≥ 150, < 225	1.762(0.872–3.559)	0.114
225–300	2.161(0.914–5.107)	0.079
> 300	2.580(0.440–15.123)	0.293
Total Gn does(IU)	0.999(0.999–1.000)	0.009
Gn Duration(days)	1.237(1.051–1.455)	0.010
E2 on HCG day(ng/ml)		
E2 < 1000		
1000 ≤ E2 ≤ 2000	1.603(0.858–2.995)	0.139
2000 < E2 ≤ 3000	1.325(0.706–2.488)	0.381
3000 < E2 ≤ 4000	1.803(0.906–3.587)	0.093
E2 > 4000	2.004(1.092–3.677)	0.025

## Discussion

The present study is the first to comprehensively explore the relationship between serum AMH levels and pregnancy outcomes in the population of Chinese individuals receiving IVF/ICSI; it is also the first to consider the factors influencing CLBR in patients with high AMH levels. The results of this study suggested that AMH levels were good predictors of CCPR and CLBR in Chinese patients undergoing IVF/ICSI. Maternal age, duration of infertility, PCOS status, bFSH levels, bLH levels, AFC levels, total Gn dose, Gn duration, serum E2, P, and LH levels on HCG day, the number of retrieved oocytes, and the number of MII oocytes were significantly different in patients with normal serum AMH levels compared to those with high serum AMH levels. There was a significant correlation between serum AMH levels and controlled ovarian stimulation outcomes.

### High AMH levels and CLBR

Compared with the normal-AMH group, the pregnancy outcomes of fresh embryo transfer in the high-AMH group were higher in the LBR and CPR groups and lower in the MR group; however, the values were not significantly different. In contrast, CLBR was significantly increased in the high-AMH group. Consistent with these results, Tal et al. showed in 2014 that increased AMH levels were correlated with higher fresh CPR^27^. A recent study suggested that higher serum AMH levels were associated with a poorer LBR in a fresh cycle^28^. However, with a larger sample size, the present study found that the LBR of the fresh cycle in the high-AMH group was improved (although the difference was not significant), and that the CLBR was significantly increased. Therefore, CLBR tended to increase with increasing AMH levels. Previous studies have disagreed regarding whether AMH levels predict oocyte quality. There are two main hypotheses on this point. The first is that AMH only affects the ovarian response^29,30^; the second is that AMH also affects oocyte quality and reproductive outcomes^31–35^. We here found that the number of retrieved oocytes and the number of MII oocytes were increased in those with higher AMH levels. In addition, as AMH levels increased, the E2 levels on HCG day increased to > 4000 ng/ml, and CLBR increased significantly. These results could thus partially explain the increased CLBR associated with increasing AMH levels. In addition, although some studies have indicated that high AMH levels may cause disturbed folliculogenesis^36–40^, others show that ovarian hyperstimulation and the associated high estrogen-induced endometrial receptivity damage do not affect the FET cycle outcome^19^. The results of the present study suggested that higher AMH levels may have a greater effect on oocyte production and quality than normal AMH levels. The possible impact of AMH on folliculogenesis and oocyte quality should be further investigated in clinical studies and in molecular biological experiments. Additional studies designed to evaluate blastocysts and Preimplantation Genetic Testing-Aneuploidy could provide further information regarding oocyte quality. The current study included a large sample size, and the main outcomes were objective indexes unaffected by subjective patient factors. Thus, the results showing significant differences can be considered highly reliable.

### High AMH levels, maternal age, and CLBR

Chinese women under age 25 tend to have significantly higher AMH levels than white women do. We here noted a positive correlation between maternal age and AMH levels in patients younger than 25 years old and a decline in AMH levels after 34 years of age until menopause. Previous studies have revealed no consistent linear relationship between age and AMH level, but that AMH decreases from age 30 until menopause^41^. The age-related decline in AMH levels is greater in Chinese women, resulting in 28% and 80% reductions in AMH levels by 30 and 45 years of age, respectively^42^. These prior results were consistent with the findings of this study. The mean age was significantly lower in the high-AMH group than in the normal-AMH group (31.67 ± 4.08 vs. 34.24 ± 4.21 years; *p* < 0.001). Previous studies have suggested that maternal age has a substantial impact on ovarian reserves. Thus, advanced age decreases oocyte production during ART cycles^43^. However, for patients with high AMH levels, ovarian reserves are generally good. It is currently unclear whether advanced age has an impact on CLBR of patients with high AMH levels^25^.The results of the present study suggested that CLBR was significantly lower in high-AMH patients over 40 years old compared with other age subgroups. In contrast to the results of this study, a study by Kai Lun Hu^44^ showed that the CLBR decreased in women younger than 35 years with serum AMH levels > 5 ng/ml (when the number of oocytes retrieved was > 20) compared with women who had lower AMH levels. Another study by Dang Kien Nguyen^20^ examined the relationship between AMH levels and the CLBR after fresh and subsequent FET during the 36-month follow-up period, and found that serum AMH levels were significantly associated with the CLBR following IVF/ICSI, independent of maternal age^20^. However, these studies failed to completely consider a variety of confounding factors that may influence CLBR, e.g., whether patients had PCOS; bFSH and bLH levels; Gn dose and duration in the ART cycle; and E2 levels on HCG day. In the present study, the AUC values of CLBR and CCPR averaged 0.664 (ranging from 0.621 and 0.706) and 0.661 (ranging from 0.613 and 0.708) (*p* < 0.001), respectively, demonstrating moderate predictive value of AMH levels for CLBR and CCPR. This study included as many potentially influencing factors as possible to reduce the bias caused by confounding factors, and showed that the CLBR was significantly higher in the high-AMH group than in the normal-AMH group. Furthermore, binomial logistic regression analysis indicated that maternal age > 40 years was significantly associated with CLBR in the high-AMH group, excluding other confounding factors. Age stratification revealed that maternal age over 40 years had a significant negative impact on CLBR compared with other age subgroups (OR = 0.137 [0.028–0.680], *p* = 0.015). These results were consistent with previous studies suggesting that advanced maternal age substantially affects CPR and LBR^45–49^. Therefore, even if the ovarian reserve was good in patients with high AMH levels, age (especially > 40) still markedly influenced CLBR. Age-related changes in oocyte quantity and quality, embryo quality, and even endometrial receptivity have a negative impact on pregnancy outcomes, thereby reducing CLBR ^50^. Consequently, patients over 40 years old seeking to become pregnant are still recommended to receive IVF treatment even if they have high AMH levels.

### High AMH levels and PCOS

An earlier study proposed a correlation between high AMH levels and adverse pregnancy outcomes in patients with PCOS. They found that increased AMH levels led to abnormal folliculogenesis, endometrial receptivity changes, and placental abnormalities^28^. In 2021, another study showed that higher baseline AMH levels in women with PCOS resulted in lower LBR and CPR, but did not influence CLBR^19^. However, the sample sizes of these studies were relatively small. Interestingly, the present study showed that significantly more patients in the high-AMH group than in the normal-AMH group had been diagnosed with PCOS (3.76% vs. 23.65%; *p* < 0.001), although a binomial logistic regression analysis indicated that whether patients with high AMH levels also had PCOS had no effect on CLBR. Using CLBR as a pregnancy outcome index may be more effective than single fresh embryo transfer in PCOS patients with high AMH levels. Because more embryos could be transferred overall when pregnancy outcomes were counted within a one-year treatment period, the impacts of the single fresh embryo transfer on endometrial receptivity due to PCOS and of high AMH levels could ultimately be minimized.

### High AMH levels and ovarian responses

A study by Tal et al.^27^ showed that increased AMH levels were correlated with greater ovarian stimulation. Consistent with previous studies^51–55^, we here found that the number of retrieved oocytes and the number of MII oocytes were significantly increased in the high-AMH group compared to the normal-AMH group. Moreover, the total Gn dose was lower in the high-AMH group than the normal-AMH group (2654.14 ± 908.48 vs. 1896.41 ± 689.52 IU; *p* < 0.001). Total gonadotrophin consumption was inversely correlated with AMH levels. These results indicated that AMH levels could positively predict ovarian responsiveness to gonadotrophin^28,56–58^.

### High AMH and E2 levels on HCG day

In the present study, E2 levels on HCG day were significantly positively correlated with serum AMH levels. However, previous studies on this topic have yielded conflicting results. In 2010, an randomized controlled trial(RCT) study of 60 patients with PCOS showed that E2 levels on HCG day were significantly negatively correlated with AMH levels among different groups (< 2.54 ng/ml, 2.54–3.85 ng/ml, and > 3.85 ng/ml AMH)^56^. In 2020, a retrospective cohort study of 184 women with PCOS undergoing their first fresh IVF/ICSI cycle found no correlation between AMH and E2 levels on HCG day^28^. However, a different study of 164 patients with PCOS suggested a positive relationship between AMH and E2 levels^57^; this is consistent with the findings of the present study, which had a larger sample size. Here, the population with high AMH levels served as the research subject, and PCOS was only one of many potential influencing factors. The range of AMH levels studied here was also broader, ranging from 1.1 and 66.6 ng/ml. Furthermore, the mechanism of interaction between AMH and E2 needed to be explored. GCs can synthesize E2 through the action of aromatase. Many studies have shown that AMH can inhibit aromatase expression, which is induced by FSH and LH in GCs, thereby significantly reducing E2 production^36,59,60^. These previous findings seem to contradict our results. However, other studies have also shown that E2 and AMH are positively correlated^57,58,61,62^. The AMH level is assumed to be lower in mature follicles than in preantral and small antral follicles, explaining this finding; it also contributes to the relief of aromatase expression inhibition and subsequent E2 synthesis^57^. AMH is only expressed by GCs with mitotic activity^60,61^. Therefore, these findings indicate that E2 may reflect GC proliferation during FSH-stimulated follicle growth, which could neutralize AMH inhibition of FSH-induced aromatase activity^58^.

## Conclusions

In this cohort study of 1379 women undergoing IVF/ICSI, we found that patients with high AMH levels had higher CPR, higher LBR, and lower MR with no statistically significant differences, although there were significant improvements in CLBR. This may have been due to the improvements in oocyte quantity and quality and changes in endometrial receptivity and homeostasis associated with high AMH levels. Advanced age (> 40 years) still impacted CLBR, even in women with good ovarian reserves. Consequently, it is still recommended that patients over 40 years old with high AMH levels actively receive IVF treatment if they seek to become pregnant. PCOS diagnoses did not influence the CLBR. In summary, this study showed that serum AMH levels could positively predict patient ovarian responses and further affect pregnancy outcomes. Larger, multicenter prospective studies and molecular experiments are required to further clarify this relationship.

## Data Availability

The datasets used and/or analysed during the current study are available from the corresponding author on reasonable request.

## Abbreviations

AMH: Anti-Müllerian hormone
LBR: Live birth rate
CLBR: Cumulative live birth rate
CCPR: Cumulative clinical pregnancy rate
CPR: Clinical pregnancy rate
IVF/ICSI: In vitro fertilization/Intracytoplasmic sperm injection
AFC: Antral follicle count
FSH: Follicle stimulating hormone
ART: Assisted reproductive technology
FET: Frozen embryo thawing transfer
E2: Estradiol
P: Progesterone
MII: Metaphase II
MR: Early miscarriage rate
PCOS: Polycystic ovarian syndrome
rHCG: Recombinant human chorionic gonadotropin
GnRH-a: Gonadotropin-releasing hormone agonist
GCs: Granulosa cells
